# Parents’ Attitudes Regarding Their Children’s Play and Sport During COVID-19

**DOI:** 10.1177/10901981221116789

**Published:** 2022-08-16

**Authors:** Monika Szpunar, Leigh M. Vanderloo, Brianne A. Bruijns, Stephanie Truelove, Shauna M. Burke, Jason Gilliland, Jennifer D. Irwin, Patricia Tucker

**Affiliations:** 1 University of Western Ontario, London, Ontario, Canada; 2 ParticipACTION, Toronto, Ontario, Canada; 3 Lawson Health Research Institute, London, Ontario, Canada

**Keywords:** physical activity, COVID-19, children, Ontario, attitude, risk tolerance

## Abstract

The COVID-19 pandemic and associated public health measures have interrupted the daily routines of parents and children. The purpose of this study was to explore parents’ attitudes regarding their children’s play/sport during COVID-19. A secondary objective was to explore the influence of parent demographics and parent-reported physical activity levels and risk tolerance on these attitudes. Ontario parents of children aged 12 and younger completed an online survey (August—December 2020) that assessed their attitudes (grouped by support, safety and socialization-related attitudes; *n* = 14 items) regarding their child(ren)’s play/sport, their physical activity levels (*n* = 2 items), and demographic details (*n* = 16 items). Two open-ended items were used to gather a deeper understanding of attitudes. Parents’ tolerance for risk was measured via the validated Tolerance of Risk in Play Scale. Descriptive statistics were calculated to describe attitudes and risk tolerance. Least Absolute Shrinkage and Selection Operator regressions were conducted to examine factors influencing parents’ attitudes. Multiple linear models were computed using the identified predictors for each attitude category. Deductive content analysis was undertaken on open-ended responses. Participants (*n* = 819) reported the highest scores for safety-related attitude items (*M* = 3.54, *SD* = .63) followed by socialization and support, which all influenced attitudes regarding children’s play/sport *(p* < .05). Demographics and parents’ physical activity levels were identified as important predictors of parents’ attitudes. Qualitative data revealed that parents had mixed levels of comfort with respect to their children’s return to play/sport. Findings from this study reveal that increased support is needed to guide future play/sport decision-making.

Physical activity during childhood offers many health benefits (Carson et al., 2017; Timmons et al., 2012). Nonetheless, since the announcement of the SARS-CoV-2 Coronavirus Disease (COVID-19) on March 11, 2020 (World Health Organization, 2021), opportunities for children’s physical activity have largely changed (Moore et al., 2020; Nigg et al., 2021; Pelletier et al., 2021). Many countries imposed physical distancing rules and contact restrictions (e.g., maintain a distance of 2 m from others; World Health Organization, 2021), and while important to protect citizens, these public health measures limited children’s opportunities to engage in activity with peers outside of their household, which has been recognized to positively influence physical activity levels (Barkley et al., 2014). Furthermore, in Canadian provinces such as Ontario, these public health measures included restrictions of settings (e.g., schools, outdoor playgrounds, and sports facilities) that previously supported children’s physical activity participation (Government of Canada, 2021). These measures have remained in place for extended periods of time, with staggered phases of re-opening across Ontario depending on contextual factors (e.g., state and risk of transmission, positive cases; Government of Ontario, n.d.). More specifically, during the timeframe of this study, citizens across Ontario were in 1 of 3 of Ontario’s phases of re-opening dependent on their city of residence; these phases directed the types of activities that were permitted (*Ontario Newsroom*, n.d.).^1^

As 2 years have passed since the onset of the pandemic (World Health Organization, 2021), studies exploring the influence of COVID-19 public health restrictions on children’s physical activity have transpired (Bates et al., 2020; Moore et al., 2020; Okely et al., 2021; Tandon et al., 2021; White et al., 2019). Most studies have identified decreases in children’s physical activity (Dunton et al., 2020; Medrano et al., 2021; Moore et al., 2020), including reduced higher intensity activity (e.g., moderate-to-vigorous physical activity [MVPA]; Tulchin-Francis et al., 2021), while some have shown no significant change (Okely et al., 2021). Furthermore, some research has highlighted a shift in children’s engagement in structured activities (e.g., sports participation) toward more unstructured or child-led play (e.g., outdoor free play; Dunton et al., 2020; Pelletier et al., 2021; Szpunar et al., 2021). However, findings are inconsistent and largely dependent on geographical location and community type (Mitra et al., 2020).

To date, factors found to be associated with increases in children’s physical activity during COVID-19 in Canada have included access to outdoor spaces (Szpunar et al., 2021), dog ownership (Moore et al., 2020), living in low-density areas (Mitra et al., 2020), and encouragement from parents/family members to engage in movement (Moore et al., 2020). Nevertheless, COVID-19 has required many to adapt the ways in which they get active (e.g., engaging at home or outdoors vs. at organized sport). This has revealed new barriers for parents such as financing new toys and activities, time constraints due to work from home and homeschooling demands, and lack of motivation (Szpunar et al., 2021).

Parent’s (including guardians) influence their children’s movement opportunities through role-modeling, providing encouragement, enrolling and paying for their children’s participation inclusive of registration and equipment fees, organizing their scheduling, and providing needed transportation (Gustafson & Rhodes, 2006; Rhodes et al., 2020; Strauss et al., 2018; Van Der Horst et al., 2007). Likewise, researchers have previously identified various demographic factors, including socioeconomic status and geographical location that influence parents’ ability to support children’s activity (Inchley et al., 2005; Smith et al., 2010). For example, Smith et al. (2010) identified cost and transport as notable barriers to supporting children’s movement, prior to a pandemic. Finally, a handful of studies have explored how parents’ risk tolerance impacts their children’s physical activity opportunities (Brussoni et al.,2021, 2018; Hilland, 2019; Jelleyman et al., 2019); for example, research has found that parental fears can diminish children’s opportunities to engage with risk (Jelleyman et al., 2019). This is an especially important consideration in the COVID-19 era.

Given the associated COVID-19 transmission risks that may be associated with allowing children to return to their pre-COVID-19 activities, some parents may be hesitant to do so. The purpose of this article was to explore parents’ attitudes regarding their children’s physical activities inclusive of play and sports during COVID-19 (e.g., as reported by participants during the timeframe of this study—August to December 2020). A secondary objective was to explore if demographic factors (e.g., housing type and the number of children) or parent-reported MVPA levels and risk tolerance influenced these attitudes. It was hypothesized that the re-opening of facilities that support physical activity would leave Ontario parents with a challenging dilemma regarding their child(ren)’s return (or not) to their previous physical activity-related programming and that parents’ attitudes would vary based on demographic and parent-reported MVPA and risk tolerance.

**Impact Statement**

This article assessed parents’ attitudes toward their children’s play/sport during the COVID-19 pandemic. Parent participants reported higher scores toward safety-related attitudes, when compared with support and socialization attitudes. Parents’ moderate-to-vigorous physical activity showed a positive, albeit nonsignificant association with safety and socialization-related attitudes.

## Method

### Study Design and Procedures

*Return to Play*, a repeated measures study that employed multiple online surveys (August—December 2020; survey 1 and August—December 2021; survey 2) using Qualtrics, assessed Ontario parents’ perspectives of their children’s (≤12 years) physical activity-related behaviors over the course of the pandemic as well as their plans for their children’s play/sport during various time points of COVID-19. The surveys asked parents to report on types of activities their child(ren) engaged in prior to COVID-19 (e.g., sports enrolled in, amount of time spent in these activities per week), spaces that children engaged in play/sport prior to and during COVID-19 (e.g., at home, at sports facility, and gyms), amount of time children spent engaged in physical activity prior to the pandemic, and parents’ perceptions of changes in their children’s physical activity levels. This article presents cross-sectional findings from Survey 1 (e.g., baseline; August—December 2020). Ethical approval was provided by the Non-Medical Research Ethics Board at the University of Western Ontario (REB #116331).

### Recruitment and Participants

English-proficient parents living in Ontario with children aged 12 years or younger (at the time of the first survey; with custody at least 50% of the time) were invited to participate. Recruitment took place using various social media platforms (e.g., Twitter), where infographics with study details (e.g., eligibility criteria, principal investigators’ contact details) were shared. In addition, various sport and physical activity organizations across Ontario were contacted and invited to circulate study details to their respective communities. Upon confirming their eligibility, participants were directed to the survey, the completion of which indicated their consent to participate.

### Instruments and Tools

The online survey (Survey #1) was created by the research team to address the overarching objectives of the Return to Play study. Survey items were informed by the COVID-19 situation in Ontario, Canada, at the time of survey creation and were tailored to encompass guidance from Ontario’s specific re-opening framework (as outlined in the summer of 2020). The baseline survey in its entirety contained 162 items; however, for the purpose of this article, a subset of 64 items across four sections (i.e., parents’ demographic characteristics [*n* = 16], parents’ MVPA levels prior to and during COVID-19 [*n* = 2], parents’ self-reported risk tolerance [*n* = 30], and their attitudes [*n* = 14] regarding their children’s play/sport programming) were examined. The validity and reliability of this study’s questionnaire have not been tested, apart from the Tolerance for Risk in Play Scale.

#### Demographic Questions

Demographic questions included parent age, number of children, the highest level of education, family situation (e.g., single parent, double parent), approximate yearly household income, and employment status.

#### Parent-Reported Physical Activity

Parent’s MVPA levels prior to and during COVID-19 were assessed via multiple-choice format (e.g., *how many minutes per week did you spend engaged in moderate-to-vigorous, heart-pumping activity prior to COVID-19?*). Parents were given 5 response options ranging from less than 30 minutes per week to more than 150 minutes per week.

#### Attitude Questions

Reported on a 5-point Likert-type scale (i.e., 1—*strongly disagree* to 5—*strongly agree*), parents were asked 14 questions about their comfort and beliefs (i.e., attitudes) regarding their child(ren)’s eventual return to various forms of play and sport during COVID-19 (e.g., *I feel willing to return my child to active play opportunities where they can follow physical distancing guidelines*). For analysis, attitude items were grouped into three thematic categories: *support-related attitudes* (items regarding parents’ skills and access to resources that support children’s play while at home during COVID-19; *n* = 6 items); *safety-related attitudes* (items pertaining to physical distancing and overall perceived safety either at home or at play/sport environments; *n* = 5 items); and *socialization-related attitudes*, items concerning their child(ren)’s socialization as a result of COVID-19; *n* = 3 items. Means and standard deviations were computed for each individual attitude, and a total mean score for each of the three attitude groups was computed.

Two-open ended questions were included to allow participants to share more in-depth responses, that is, “Please describe the reasons you do or do not feel comfortable with the idea of your child(ren) returning to their active play/sports activities that they engaged in prior to COVID-19”; “To help increase our understanding, please explain your plan to return your child(ren) to their active play/sport programming (e.g., how you are [or not] planning to return your child(ren) to activities they engaged in prior to COVID-19).”

#### Tolerance of Risk in Play Scale

A modified version (i.e., 30 of 32 items) of the Tolerance of Risk in Play Scale (TRiPS; Hill & Bundy, 2014) was included at the end of the survey to assess parents’ self-reported risk tolerance for engaging children in various types of play (e.g., playing outdoors while unsupervised). Two items from the scale were removed as they were not appropriate for the age group (i.e., some parents responding for children under 3 years of age). This valid and reliable survey (i.e., Pearson reliability index of 0.87) was originally developed to assess adults’ tolerance for outdoor risky play among children aged 3 to 13 years (Hill & Bundy, 2014), and its psychometric properties have been previously demonstrated with parents and elementary school teachers as respondents (Brussoni et al., 2018). The first item of the tool asks participants, “How often do you encourage everyday risks?” with four response options, ranging from “never” to “often,” while all remaining items provide a “yes” or “no” response. Raw scores were collected (i.e., *yes* = 1; *no* = 0), and per the tool creator’s recommendation, a higher total score indicating greater tolerance for risk during children’s play.

### Data Preparation and Analysis

Descriptive statistics were used to report parent demographics and risk tolerance and were computed in SPSS (version 27). All other data preparation (i.e., data cleaning) and analyses were computed using R statistical software, version 3.6.1 (R Core Team, 2019). Survey responses with more than 15% missingness (i.e., incomplete data) were removed (Li et al., 2020). A self-defined function was employed to insert median values in instances where a small number of participants did not complete a demographic question (e.g., provided parent age but not ethnicity), and imputation methods were applied to address variables with high levels of missing data using the K Nearest Neighbor (KNN) algorithm (Zhang, 2016). Because some parents provided responses for multiple children, children’s biological sex and age were concluded across families to provide family indices.

Three Least Absolute Shrinkage and Selection Operator (LASSO) regressions (one for each attitude category) were conducted to identify whether demographic and/or parent-reported physical activity and risk tolerance influenced parents’ attitudes regarding their children’s play/sport during and following COVID-19. LASSO regressions reduce unstable estimates to zero to exclude variables without the need for formal statistical testing and are frequently used when a large number of covariates need to be considered (Steyerberg et al., 2001). Once predictors were identified from the LASSO regression, multiple linear models (R package “*lmtest*”) were computed for each attitude category; however, upon visual inspection of the QQ plots for safety and socialization-related models, nonappropriate shapes were observed and the Shapiro tests reported very small *p* values (*p* = 1.80 and 1.90, respectively). Consequently, Box-Cox transformations (R package “*EnvStats*”; Millard & Kowarik, 2022) were undertaken to improve the linear models and normalize the residuals to increase the applicability and usefulness of the data for the safety and socialization models (Teugels & Vanroelen, 2004). Parameter lambdas (λ) were set to 2 based on minimizing the log-likelihood of potential models for the safety and socialization-related attitude models. *F*-tests were used to identify if categorical variables were significant. The two open-ended questions were analyzed via deductive content analysis (Kyngäs & Kaakinen, 2020) by two independent researchers using QSR NVivo (Version 12), and common responses were identified based on the questions asked (e.g., assessing plan to return and comfort to return; Anderson, 2010).

## Results

A total of 819 parents, mean age of 38.1 (*SD* = 6.1) years, participated; the majority identified as female (93%), Caucasian (84%), lived in a detached house (77%), and had full-time employment (65%). Most parents (59%) had 2 or more children, and children were, on average, 6.5 (*SD* = 3.2) years of age, and most were reported to be female (69%). Participants most frequently (27.4%) reported spending less than 30 min/week in MVPA during the pandemic. The average risk tolerance score was 17.1 (*SD* = 5.1) out of 29, and most participants (56%) reported *sometime*s encouraging children’s everyday risks. See Table 1 for complete parent demographics, physical activity, and risk tolerance.

**Table 1.** Parent (*n* = 819) Demographics, Self-Reported Physical Activity, and Risk Tolerance.

Demographic factors	*M*	*SD*
Parent age (years)	38.1	6.1
Child age (years)	6.5	3.2
	*N*	%
Parent gender		
Male	53	6.5
Female	762	93.0
Prefer not to say	4	0.5
Children’s biological sex		
Male	257	31.3
Female	562	68.7
Type of living		
Rural	232	28.3
Suburban	359	43.8
Urban	228	27.8
Ethnicity		
Caucasian	688	84.0
African Canadian	3	0.4
South or East Asian	51	6.2
Middle Eastern	5	0.6
Aboriginal	26	3.2
Latin American	6	0.7
Other	23	2.8
Employment status		
Full-time	536	65.4
Part-time	105	12.8
Occasional/support	30	3.7
Unemployed	109	13.3
Family situation		
Single parent	90	11.0
Double parent	703	85.8
Guardian-led	6	0.7
Other	13	1.6
Highest level of education		
High school	59	7.2
College	239	29.2
University	304	37.1
Graduate school	210	25.6
Housing type		
Apartment	46	5.6
Condo	15	1.8
Townhouse	55	6.8
Semi-detached house	59	8.3
Detached house	632	77.2
Other	12	1.5
Family dog		
Yes	363	44.3
No	456	55.7
Household income		
<$20,000	21	2.6
$20,000–$59,999	109	13.3
	*N*	%
$60,000–$99,999	174	21.2
$100,000–$139,999	194	23.7
≥$140,000	249	30.4
Number of children		
1	337	41.1
2	360	44.0
3 or more	122	14.8
Phase of re-opening		
Phase 1	29	3.5
Phase 2	93	11.4
Phase 3	697	85.1
Children’s age minimum		
0–3 years	298	36.4
3.5–6 years	212	25.9
>6.5 years	309	37.7
Children’s age maximum		
<3 years	124	15.1
3–6 years	193	23.6
>6 years	502	61.3
Parent physical activity and risk tolerance	*N*	%
Minutes/week spent in MVPA prior to COVID-19		
<30 minutes	146	17.8
30–59 minutes	180	22.0
60–89 minutes	132	16.1
90–119 minutes	104	12.7
120–140 minutes	78	9.5
150 minutes or more	179	21.9
Minutes/week spent in MVPA during COVID-19		
<30 minutes	224	27.4
30–59 minutes	171	20.9
60–89 minutes	123	15.0
90–119 minutes	86	10.5
120–140 minutes	57	7.0
150 minutes or more	158	19.3
Frequency parent/guardian encourages everyday risks		
Never	16	2.0
Seldom	99	12.1
Sometimes	456	55.7
Often	248	30.3
	*M*	*SD*
Risk tolerance	17.1	5.1

*Note*. Risk tolerance refers to parents’ raw risk tolerance score out of 29 as measured via the Tolerance of Risk in Play Scale (Hill & Bundy, 2014). Phase re-opening refers to Ontario’s re-opening framework at the time of survey completion (Government of Ontario, n.d.). Number of children refers to the number of children younger than 12 years of age that the participant legally provided care for at the time of survey completion. Children’s Age Minimum refers to the age of the youngest child within the family. Children’s Age Maximum refers to the age of the eldest child within the family. MVPA = moderate-to-vigorous-intensity physical activity. $ refers to Canadian currency.

### Summary of Parents’ Support, Safety, and Socialization-Related Attitude Scores

Parents, on average, reported slightly higher attitudes to safety-related items (*M* = 3.54, *SD* = 0.63) compared to socialization- and support-related attitude items (*M* = 3.50, *SD* = 0.54; *M* = 3.30; *SD* = 0.82, respectively). With regard to individual attitude items, “I reserve time out of my day to support my child’s active play” was ranked highest (*M* = 3.89, *SD* = 1.09) in the support category; “I feel that having my child at home with me during the pandemic makes me feel safe” (*M* = 3.86, *SD* = 1.11) in the safety category; and “I am looking forward to allowing my child to interact with others” (*M* = 4.36, *SD* = 0.93) in the socialization category. Finally, a high score was noted for the socialization item, “I feel that my child has missed out on health benefits of extracurricular activities due to the COVID-19 pandemic” (*M* = 4.19; *SD* = 1.06). See Table 2 for support-, safety-, and socialization-related attitudes and total scores for each category.

**Table 2.** Means and Standard Deviations for Parents’ Attitude Scores.

Support-related attitudes	*M*	*SD*
I have enough skills to support my child’s active play at home	3.38	1.25
I have access to what I need at home to support my child’s active play	3.38	1.18
I have the ability to support my child’s physical activity/active play at home without engagement in extracurricular activities	3.06	1.28
I have enough access to resources (i.e., space, time, toys) that allow me to support my child’s active play	3.50	1.23
I reserve time out of my day to support my child’s active play	3.89	1.09
I feel worried that I will no longer be able to afford my child’s extracurricular activities postpandemic	2.61	1.37
TOTAL support score	3.30	0.82
Safety-related attitudes	*M*	*SD*
I feel willing to return my child to active play opportunities where they can follow physical distancing guidelines	3.77	1.27
I feel that having my child at home with me during the pandemic makes me feel safe	3.86	1.11
I feel that having my child at home with me during the pandemic makes them feel safe	3.76	1.12
Even if my child can follow physical distancing guidelines, I am still hesitant to return them to active play programming	3.08	1.33
I am confident that if I return my child to active play, my child will follow Ontario’s public health guidelines (e.g., hand sanitizing)	3.24	1.30
TOTAL safety score	3.54	0.63
Socialization-related attitudes	*M*	*SD*
I am looking forward to allowing my child to interact with others	4.36	.932
I prefer to allow my child to interact with people via social networking sites and screen-based technology than in person	1.96	1.14
My child has missed out on health benefits of extracurricular activities due to the COVID-19 pandemic	4.19	1.06
TOTAL socialization score	3.50	0.58

*Note*. Each item was ranked on a 5-point Likert-type scale.

### Identified Predictors and the Influence of Demographic and Parental Factors on Parents’ Support, Safety, and Socialization Attitudes

The variable selection from the LASSO regression revealed 20 main predictors for parents’ attitudes regarding their children’s play/sport during COVID-19. See Table 3 for all identified predictors as identified by the LASSO regression for each attitude group.

**Table 3.** Main Predictors for Parents’ Safety, Socialization, and Support-Related Attitudes.

Category	Variable	Details
Demographic	Parent gender^δ^	Parent gender at measurement time
	Parent age^δ^	Parent age at measurement time
	Community type^δ,ξ^	3-response option item: “Rural”, “Urban”, “Suburban”
	Child biological sex^δ,ξ^	Parents report of their child(ren)’s biological sex
	Child age max^ξ, λ^	The age of the eldest child within the family
	Child age min^δ^	The age of the youngest child within the family
	Child age mean^ξ^	Mean age of children included in study
	Ethnicity^δ^	9-response option item: “Caucasian”, “African Canadian”, “South Asian”, “East Asian”, “Middle Eastern”, “First Nations/Aboriginal”, “Latin American”, “Other”, “Prefer not to answer”
	Employment status^δ^	5-response option item: “Full-time”, “Part-time”, “Occasional/Support”, “Unemployed”, “Prefer not to answer”
	Family situation^δ,λ^	5-response option item: “Single-parent”, “Double-parent”, “Guardian-led”, “Other”, “Prefer not to answer”
	Education level^δ,λ^	5-response option item: “High school”, “College”, “University”, “Graduate school”, “Prefer not to answer”
	Housing type^δ,λ^	6-response option item: “Apartment”, “Condominium”, “Townhouse”, “Semi-detached house”, “Detached house”, “Other housing”
	Family dog^δ,ξ,λ^	2-response option item: “Yes”, “No”
	Household income^δ^	9-response option item: “less than $20,000”, “$20,000-$39,000”, “$40,000-$59,000”, $60,000-$79,000”, $80,000-$99,000”, $100,000-$119,000”, $120,000-$139,000”, “more than $140,000”, “prefer not to answer”
	Phase re-opening^δ,λ^	Phase re-opening at time of survey completion. 3-response option item “Phase 1”, “Phase 2”, “Phase 3”
	Number of children^δ,λ^	Number of children at time of survey completion
Parental	Parents self-report moderate-to-vigorous physical activity level before COVID-19^δ^	6-point item: “Less than 30 minutes”, “30–59 minutes”, “60–89 minutes”, “90–119 minutes”, “120–149 minutes”, “150 minutes or more”
	Parents self-report moderate-to-vigorous physical activity level during COVID-19^δ,ξ,λ^	6-point item: “Less than 30 minutes”, “30–59 minutes”, “60–89 minutes”, “90–119 minutes”, “120–149 minutes”, “150 minutes or more”
	How often do you encourage your child to take everyday risks^δ,ξ,λ^	4-point item: “Never”, “Seldom”, “Sometimes”, “Often”
	Overall risk tolerance^δ,ξ^	Total yes score out of 29 “yes” or “no” questions

*Note*. Phase re-opening refers to Ontario’s re-opening framework as outlined by the Ontario Government in 2020 (Government of Ontario, n.d.). Overall risk tolerance was assessed using a modified version of the validated TRiPS tool (Hill & Bundy, 2014).

δ,ξ,λ = variables selected for the final model by LASSO regression for support-related factors, safety-related factors, and socialization-related attitudes, respectively.

#### Support-Related Attitudes

For support-related attitudes, significant predictor variables were parent age, living in an urban community, family situation (i.e., in the “other” category), and not having a family dog. This model accounted for 17% of the variability observed, *F*(58, 713) = 3.76, *p* < .*001*. See Table 4 for complete results from the mixed linear models exploring various predictors’ influences on support-related attitudes.

**Table 4.** Influence of Demographic and Parental Factors on Support-, Safety-, and Socialization-Related Attitudes—Mixed Linear Model Results.

Variable	Support			Safety			Socialization		
	Estimate	*SE*	*p*-value	Estimate	*SE*	*p*-value	Estimate	*SE*	*p*-value
Community type									
Urban	−.18	.07	.017*	.59	.18	.001**			
Suburban	−.06	.06	0.33	.19	.20	.35			
Parent gender									
Female	.01	.11	.96						
Prefer not to answer	.61	.51	.23						
Parent age	−.01	.01	.017*						
Ethnicity									
African Canadian	.12	.42	.77						
South Asian	−.20	.14	.13						
East Asian	−.17	.19	.38						
Middle Eastern	.06	.37	.88						
Aboriginal	.44	.17	.008**						
Latin American	−.14	.32	.67						
Other	.14	.16	.37						
Prefer not to answer	.13	.24	.60						
Employment status									
Part time	.32	.08	7.84***						
Occupational/support	.23	.14	.10						
Unemployed	.28	.09	.001**						
Prefer not to answer	039	.14	.005**						
Family situation									
Double parent	.02	.10	.80				−.01	.21	.98
Guardian-led	−.06	.34	.85				−.82	1.09	.45
Other	.50	.23	.027*				−1.19	.61	.05
Prefer not to answer	.06	.38	.88				−.07	.89	.93
Highest level of education									
College	−.02	.12	.86				−.20	.28	.48
University	−.05	.12	.66				−.18	.28	.51
Graduate school	.02	.13	.89				−.42	.29	.16
Prefer not to answer	−.11	.47	.82				−1.15	1.01	.25
Housing type	.14	.25	.58				.82	2.68	.76
Condominium									
Townhouse	−.15	.16	.34				.37	1.13	.74
Semi-detached house	.20	.15	.19				.59	1.08	.59
Detached house	.18	.13	.16				−.01	.79	.99
Other	0.01	0.26	0.96				−3.09	2.28	.18
No family dog	.14	.06	.014*	0.25	0.15	.10	−.16	.13	.23
Household income	−.13	.20	.52						
$20,000–$39,999									
$40,000–$59,999	.08	.21	.70						
$60,000–$79,999	.07	.20	.72						
$80,000–$99,999	.09	.20	.66						
$100,000–$119,999	−.05	.21	.81						
$120,000–$139,999	−.14	.21	.50						
≥$140,000	−.04	.20	.86						
Prefer not to answer	.25	.21	.24						
Phase re-opening									
2	−.12	.16	.45				.95	.43	.028*
3	−.13	.15	.37				.20	.39	.60
Number of children	−.05	.04	.17				−.58	.46	.21
Parents PA before COVID									
30–59 min/week	.06	.09	.49						
60–89 min/week	.02	.09	.81						
90–119 min/week	.09	.10	.41						
120–149 min/week	−.02	.12	.83						
≥150 min/week	.14	.10	.17						
Parents PA during COVID				.02	.21	.94	−.40	.18	.029*
30–59 min/week	.12	.08	.12						
60–89 min/week	.25	.09	.01**	.03	.23	.88	−.24	.20	.24
90–119 min/week	.32	.10	.002**	.38	.26	.14	−.33	.23	.16
120–149 min/week	.48	.12	.001**	−.11	.31	.72	−.62	.27	.022*
≥150 min/week	.42	.10	.001**	.59	.22	.006**	−.49	.19	.011*
How often parent encourages risks									
Seldom	–.25	.22	.24	−.70	.57	.22	−.24	.56	.66
Sometimes	–.07	.21	.730	−0.70	0.54	0.20	−.41	.53	.45
Often	.08	.21	.71	−0.93	0.56	0.10	−.69	.54	.21
Parents risk tolerance	–.00	.01	.92	−.03	.02	.10			
Children’s age minimum	−.03	.01	.008**	.04	.06	.51			
Children’s age maximum				.03	.06	.62	.26	.09	.005**
Child sex	.02	.07	.79	.51	.20	.01**			
Interaction effect housing type—condominium and child age maximum							−.47	.22	.04*
Interaction effect housing type—townhouse and child age maximum							−.04	.12	.72
Interaction effect housing type—semi-detached house and child age maximum							−.24	.12	.05*
Interaction effect housing type—detached house and child age maximum							−.16	.09	.10
Interaction effect housing type—other and child age maximum							−.15	.28	.59
Interaction effect housing type—condominium and number of children							1.58	1.43	.27
Interaction effect housing type—townhouse and number of children							.07	.58	.90
Interaction effect housing type—semi-detached house and number of children							.52	.61	.39
Interaction effect housing type—detached house and number of children							.48	.47	.31
Interaction effect housing type—other and number of children							1.87	.73	.01*

*Note*. Risk tolerance refers to parents’ raw risk tolerance score as measured via the Tolerance of Risk in Play Scale (Hill & Bundy, 2014). Phase re-opening refers to Ontario’s re-opening framework at the time of survey completion (Government of Ontario, n.d.). Children’s age min refers to the age of the youngest child within the family. Children’s age max refers to the age of the eldest child within the family. Child sex refers to the proportion of female children within the family. MVPA = moderate-to-vigorous-intensity physical activity. Safety and socialization results are presented from the adjusted models. The support model accounted for 17% of the variability observed, *F*(58, 713) = 3.76, *p < .001*. The safety model accounted for 4% of the variability observed, *F*(15, 803) = 3.59, *p* < .001. The socialization model accounted for 7% of the variability observed, *F*(36, 726) = 2.63, *p < .001*.

* *p* ≤.05. **p ≤ .01. ***p ≤ 0.001.

#### Safety-Related Attitudes

No variables showed a significant association with safety-related attitudes. Living in a suburban environment, risk tolerance, children’s biological sex, and parents who engaged in more than 150 min/week of physical activity prior to COVID-19 all approached significance with safety-related attitudes (*p* < .10). This model accounted for 4% of the variability observed, *F*(5, 803) = 3.59, *p* < .001 (see Table 4).

#### Socialization-Related Attitudes

Being in Phase 2 of Ontario’s re-opening plan at the time of survey completion, children’s age maximum (i.e., having older children), physical activity levels of parents during COVID-19 (i.e., specifically those with higher levels of weekly MVPA), and the interaction between housing type and age of children, and housing type and the number of children had a significant effect on socialization-related attitudes (*p* < .05). The model accounted for 7% of the variability observed, *F*(36, 726) = 2.63, *p < .001* (see Table 4).

### Parents’ Level of Comfort and Plans to Return Their Child(ren) to Play/Sport

Regarding parents’ level of comfort returning children to play/sport, there was a range of views. The most common parent reasons for feeling comfortable included: if it was deemed safe per COVID-19-related public health guidelines in Ontario; low case numbers in their respective communities; a desire to return to normalcy and routine; comfort to return to outdoor activities only; and to support the physical and mental health of their children. Reasons for not feeling comfortable included children being too young to properly follow and understand public health guidelines (e.g., distancing); fear of others (e.g., other children and other families) not following guidelines and consequently spreading the virus; concern for vulnerable children or other family members; the uncertainty of facilities’ abilities to uphold safety protocols (including physical distancing); and increased costs of activities and general financial strain associated with the pandemic. Similar themes were noted regarding parents’ plans to return their children to play/sport (e.g., plan to return to outdoor activities only, plan to return if deemed safe by public health guidelines, etc.) with the addition of “plan to return only once there is a vaccine available.” See Table 5 for themes, subthemes, and supporting quotes concerning parents’ level of comfort and plans for their child(ren)’s return to play/sport.

**Table 5.** Parents Level of Comfort Returning Their Child(ren) to Play/Sport and Plans to Return.

Theme	Example quotes by subtheme
Comfortable to return child(ren) to play and/or sport	Comfortable to return if deemed safe by public health guidelines – “I feel comfortable because I trust our health teams and government policies surrounding COVID-19.” – “I feel comfortable as long as the correct COVID protocols are in place.” – “Most sports are taking extensive measures to ensure physical distancing and safety of all participants (more so than school!).” – “I feel comfortable with my children returning to track and field because the track team has a solid return to play guideline following the guidance of Athletes Canada and Athletics Ontario.” – “I feel most comfortable about them returning to hockey because hockey Canada is providing associations with guidelines and support that help everyone follow recommended guidelines.”
	Comfortable to return given low case counts/community transmission – “I feel mostly comfortable as the cases are very low in our area and the places my kids go are doing a good job addressing the situation.” – “I am comfortable because we live in a small rural area, where we pretty much [all] know each other.” – “We live in a very small community with no active cases of COVID so as long as it stays that way, I am completely fine with my kids returning to play/sport activities.”
	Comfortable to return given the additional physical and mental health benefits – “The mental health of the children is being affected.” – “I worry about the damage to my children’s physical and emotional health if we don’t.” – “I believe keeping children from participating in extracurricular activities may cause as much or more long-term damage than COVID-19.” – “Children need to play and. . . interact and learn with and from other children. The reward is greater than the risk of contracting COVID-19.” – “I feel comfortable because of the immediate positive impact I have witnessed with returning back to both school and recreational activities on his mental health and social well-being. My son is physically and mentally happier, engaged, focused and positive about life and I believe that a child needs these outlets to grow and build self-confidence and resiliency.”
	Comfortable to return because of the desire to return to normalcy and have routines – “I feel he needs to get back into a regular routine and interact with other children and authority figures other than his parents at home.” – “I think we all need to get back to a normal life. Kids need to socialize.” – “They need social interaction and a return to their “normal” activities that cannot be duplicated at home.”
Not comfortable to return child(ren) to play and/or sport	Not comfortable given child’s age/inability to follow safety measures – “My children are too young to understand the importance of physical distancing and so will be at risk of contracting and spreading the virus.” – “Physical distancing is the major issue with small children—no sense of personal space. Difficult to manage.” – “We feel uncomfortable with our children returning to pre-covid indoor activities as they are too young to maintain physically distance or wear masks.” – “I’m not confident in the abilities of my children and their peers to maintain safe distances while engaging in activities.”
	Uncomfortable because of others – “I do not feel comfortable that other participants and families are taking the same precautions as my family and could potentially cause my child and family to become sick.” – “I feel it is too risky to trust others to socially distance yourself the level we are comfortable with.” – “We are very careful but are worried about other people not taking it seriously. If everyone was taking it seriously, we would be a lot more comfortable.” – “I see other parents at the places not wearing masks themselves and it makes me question how safe their family is with the safety precautions” – “I have lost a lot of trust in the public and will not be able to tell the level of caution of other people involved in the activity.”
	**Uncomfortable due to health conditions in family members** – “We have risk family member in the home, one of which is my youngest child.”‘ – “I worry about spreading the virus to people who are immune-compromised such as older family members or friends who are cancer survivors.” – My son has been diagnosed with asthma so we are quite hesitant putting him into things where he might come in contact with COVID, and what that could look like for him.” – “Fear of COVID showing no symptoms and [kids] bringing it home to loved ones who are immune-compromised, and several are elderly.” – “If my kids contract the virus on the public playground, they will bring that back home and possibly infect my husband who is diabetic, that outcome could be negative for him.”
	Not comfortable given uncertainty to uphold safety protocols (including physical distancing) – “I am also concerned about shared equipment in gymnastics, and the level of touching that would be required if I sign my 3-year-old up for swimming.” – “I am not sure some spaces are safe, despite all the efforts, to guarantee physical distance.” – “The largest concern is the ability for programmers to maintain physical distancing.” – “I worry about cleaning protocols and air ventilation in the centre.”
	Uncomfortable because lack of information guidance from sport organizations – “The guidelines for keeping kids safe (all of us safe) are constantly changing, I don’t feel we have a proper understanding of how to truly protect ourselves.” – “Don’t trust all organizers to take precautions as seriously as they should.” – “Many groups who run these activities around us are acting unconcerned about distancing and are not following the guidelines.” – “I am concerned about the abundance of rules in hockey. That play will be so restricted they won’t get the physical activity release and enjoyment that we sign them up for.” – “We feel that with all of the new rules and restrictions in place at these programs, the activities themselves will be a lot less fun than they were pre-COVID.”
	Not comfortable to return given additional costs associated with pandemic – “We are also being mindful of expenses in the event that my husband or I lose a job as the result of an economic downturn.” – “With the uncertainty of whether there will be a second wave, I am not willing to risk spending the money on activities.” – “We spend a lot of money on the sport, and I want to be able to watch my children play. That doesn’t sound like an option at this point.” – “I feel less comfortable about the financial burden though since my husband is self-employed and we took a huge hit financially this year while unable to work during the pandemic.” – “We lost a large amount of money due to the shutdown of activities when COVID-19 started. With the uncertainty of whether there will be a second wave, I am not willing to risk spending the money on activities.” – “Our family can’t afford sports equipment/fees/fundraising.”
Plan to return children to play and/or sport	Plan to return if public health guidelines are in place – “We plan to allow them to return as long as our local health unit determines it is safe to do so.” – “My children will be returning to activities that are following health unit (i.e., physical distancing) protocols.” – “Returning will be dependent on what the programming looks like and the ability to follow public health recommendations.” – “If social distancing, hand washing and masks will be enforced, I would like to enroll my children in the same activities they participated in prior to COVID.” – “Returning to hockey with proper protocol. Dressing at home, limited time in change room, wearing mask until on ice, practicing physical distancing with all screening procedures beforehand. No contact sports.”
Plan to return children to play and/or sport with some modifications	Plan to return to outdoor activities only – “We have enrolled in forest school for solely outdoor activity where natural distancing occurs.” – “We will likely not be returning to dance until physical distancing needs are gone or dramatically reduced. We will look for more outdoor activities, and activities they can do safely.” – “We will play at parks, play in the forest, go for hikes, make our own backyard ice for skating or use the outdoor skating trail. We will not participate in indoor activities.” – “We’re planning to continue with outdoors activities wherever possible throughout the pandemic.”
	Plan to return once there is a vaccine – “Activities are on hold until a vaccine becomes available and is proven safe.” – “I am uncomfortable returning my child to their previous programming until there is a vaccine available or COVID cases have stopped in our area.” – “I will allow my children to return to their activities once provincial COVID numbers drop drastically, and a safe reliable vaccine is available.”
	Plan to return them to different activities from pre-COVID – “Looking for new alternatives and wait to see how a season goes before we go back.” – “Since they’ve been away from activities for so long, I might be looking for new activities for them.” – “I expect that by the time things open they will be interested in different things as they are so young now.” “My child was in dance but has decided not to dance this year. Nothing to do with COVID.”
Do not plan to return or do not have the means to return child(ren) to play/sport	Unable to return due to lack of programming and/or finances – “Our community is very small, so we might be limited in what is offered. As of right now, there is no programming offered.” – “We live in a smaller community, so it requires lots of volunteers to make things work. Some activities have been completely cancelled (i.e., indoor soccer) but others are slowly starting up!” – “My youngest would love to do so many things that we can’t afford!” – “Our decision to return to play will be influenced by our parental ability to support participation the program (e.g., equipment, cost, time, travel, health measures, etc. . . as well as the availability and variety of programs within our neighbourhood and municipality.”
	Do not plan to return. Waiting until it is safer and engaging at home in the meantime. – “We have decoded to do things at home. Backyard gym, nature walks and bike rides.” – “My kids will do only those activities which can be done at home or in my neighborhood in my supervision.” – “My child will not be enrolled in any pre COVID-19 activities or any new ones.” – “We will not return our children to school or scheduled activities with the current health measure in place. Masking, social distancing, and extra hand hygiene are all shown to be negative to children’s social, psychological, and physical development.” – “At this point, my children will not return to organized sports or active play engaged with children outside of our family.”

## Discussion

The COVID-19 pandemic has led to extended public health measures in Ontario, Canada, as a means of limiting the spread of the infectious virus. Given the important association between physical activity-related activities (e.g., play and sport), and children’s overall health (Carson et al., 2017), the purpose of this study was to explore parents’ attitudes regarding their children’s return to these activities. Furthermore, it was examined whether demographic factors and/or parent-reported physical activity and risk tolerance influenced attitudes.

Overall, parents reported higher scores toward taking time out of their day to support their children’s physical activity (i.e., reported via support-related attitude). This is important, as prepandemic research supports the association between parents’ role-modeling and support on children’s likelihood to engage in healthy movement behaviors (Garriguet et al., 2017). For example, research conducted by Jago et al. (2011) found that higher levels of parental support with regard to promoting children’s physical activity (e.g., providing transportation) was related to greater physical activity among children (Jago et al., 2011). This is one of the first studies, to our knowledge, that explores the influence of parents’ support-related attitudes toward their child(ren)’s play/sport during the COVID-19 pandemic. Parental attitudes for supporting children’s activity during times when there is limited access to physical activity-supporting environments (e.g., gyms, sports) are important to consider.

Parents in the present study reported being eager to allow their child(ren) to interact with others (i.e., reported via socialization-related attitudes and open-ended responses). This may imply that some parents see value in the benefits of play/sport activities and their child(ren)’s socialization with others. It could also be an indication that parents need relief from parenting and homeschooling demands, as this has been previously identified by parents in Ontario during the pandemic (Szpunar et al., 2021). Allowing children to play with others during the pandemic may lead to higher levels of child-led, unstructured play, as this behavior has been found to have increased during COVID-19 (Dunton et al., 2020; Pelletier et al., 2021). The inclination reported by parents to return children to play/sport in the present study may have been due to the timeframe in which this study was conducted, with most participants in Phase 3 of re-opening (i.e., pre-Delta and Omicron waves; Public Health Ontario, n.d.).

Parents agreed that they felt their child(ren) had missed out on important health benefits associated with extracurricular activities due to staying at home for extended periods of time amid the pandemic (i.e., as reported via safety-related attitude). Furthermore, some qualitative findings from the present study revealed that parents felt they would like to return their children to play and sport programming as soon as possible. Compared with other periods where children may be home for extended periods (e.g., summer holidays from school, winter holiday break), public health measures because of COVID-19 may pose additional challenges for families as children are unable to play with others or engage in activity outside of the home as they typically would do during pre-COVID-19 extended stay-at-home periods (e.g., in the context of time away from school). Increased research is needed regarding the types of supports that can be put in place for families with children during times of extended stay-at-home periods, specifically when public health measures (e.g., physical distancing rules) are in place.

Risk tolerance was not significant regarding its influence on participants’ safety-related attitudes. This was contrary to expectations, as researchers have found that parental attitudes (e.g., fears, intentions), most frequently those of mothers, are a primary obstacle to child(ren)’s engagement in activities that involve risk (e.g., climbing a tree, playing without supervision; Boxberger & Reimers, 2019; Lee et al., 2015). However, given the TRiPS tool is a general risk tolerance scale, not one specifically designed for COVID-19 (or any pandemic), it is possible that risk was being influenced by factors that were not captured on this tool. Furthermore, parents in this study expressed that having their children at home with them during the pandemic made them feel safe. It is important to note that at the time of data collection for this study, there was no vaccine approved for children younger than 12 years of age in Canada, and many adults were not yet vaccinated (apart from front line and health care workers). Thus, it is possible that parents felt fear, or increased worry about the risk of transmission, and risk of their children contracting COVID-19 at the time of completing the survey and preferred to have them isolated at home with minimal contact. All these considerations may have led to the highest attitudes reported by participants in the present study being found for safety-related attitude items. These findings were emphasized via open-ended items, where some participants noted they did not feel comfortable or did not plan to return their children due to fears of others (i.e., other children and other families) not following proper health protocols, and because of the uncertainty of organizations/facilities being able to uphold safety protocols (and facilitate social distancing). In addition, some parents reported they were not yet willing to return their children to play/sport until a vaccine became available. Although risk tolerance did not influence support or socialization-related attitudes, we hypothesize this as a consequence of COVID-19 being more closely aligned with one’s perception of safety than their ability to support their children’s physical activity or socialization opportunities.

The regression modeling identified a number of predictors (i.e., housing type, child(ren)’s age, parent’s physical activity levels, etc.) that significantly influenced parents’ attitudes for their children’s play/sport. For example, parents’ levels of MVPA during COVID-19 influenced all three attitude categories, with those more active having more positive attitudes, specifically in the socialization category. In addition, community type, specifically living in an urban community, had a significant influence on safety attitudes. Previous research exploring the influence of community type and dog ownership on children’s physical activity levels identified that children living in rural communities and ownership of a dog had higher levels of physical activity compared to those living in urban areas, and those without a dog (Moore et al., 2020; Zenic et al., 2020); thus, our finding that living in an urban environment and not having a dog was an important predictor for safety-related attitudes was surprising and contradicts previous findings. Finally, the influence of community is important to note, as de Lannoy and colleagues (2020) found that compared to other regions in Canada, Ontario experienced the greatest decline in children’s time spent outdoors and in outdoor play compared with other provinces (de Lannoy et al., 2020). This can likely be attributed to the fact that Ontario was one of the hardest hit provinces by COVID-19 at the time of data collection, alongside Quebec, which both had the largest case counts and strictest restrictions (de Lannoy et al., 2020). In addition, because Canada has four seasons, rates of play/sport may have been influenced by environmental factors such as weather (i.e., lower physical activity levels in winter because of inclement weather, and higher physical activity during summer; Tucker et al., 2009), although this was not explored in the present study.

It is imperative that researchers further explore the effects of the pandemic on children’s future physical activity-related behaviors and the challenging decisions parents need to make with returning their children to these activities (or not). With such a small percentage of children meeting physical activity guidelines during the pandemic (Moore et al., 2020), there is a larger risk posed for developing chronic health conditions in later life. As COVID-19 continues to spread, with new variants of concern, increased support for at-home and contactless activity is needed. In addition, consideration for overcoming barriers/concerns raised by parents to support children’s physical activity participation is warranted.

### Limitations

Strengths of the present study include the early launch of the baseline survey at the start of COVID-19, the large sample of Ontario parents, the inclusion of both quantitative and qualitative data, and the use of LASSO modeling to identify predictors of parents’ attitudes. Despite noted strengths, there are also some limitations of note. First, although our efforts to recruit as many Ontario parents as possible, only 819 participants completed the survey in its entirety. In addition, the length of the survey and the participant burden related to completion time may have led to incomplete data from some participants. Moreover, the survey respondents were primarily female, Caucasian, double parent, with higher average household incomes, which limits the generalizability of the findings. Finally, the lack of validity and reliability of the survey (with the exception of the Tolerance of Risk in Play Scale; Hill & Bundy, 2014) due to its creation by the research team and self-report nature of the survey also acts as a limitation. Given the responses were self-reported by participants, responses may have been influenced by social desirability bias (i.e., parents may have felt pressured to select a more desirable option for some questions). Finally, because this study took place during early COVID-19 when vaccines were not yet widely available, the survey did not capture the vaccination status of participants, or the consideration this might have for parents play/sport decision-making. In addition, it is important to note that several factors beyond those that were explored in the present study could have influenced parents’ attitudes of children’s play/sport, including but not limited to case counts in participants’ communities.

## Conclusion

This study identified Ontario parents’ support, safety, and socialization-related attitudes toward their children’s return to play/sport during COVID-19. Parents had the highest attitudes toward safety-related items. In addition, important predictors of parents’ attitudes were highlighted, such as the influence of parents’ MVPA levels on attitudes. An equal divide was found between Ontario parents who feel comfortable returning their children to sport and those who do not. Future investigations are needed to explore what types of supports are needed to ensure children’s seamless transition back into physical activity-supportive environments (e.g., sport arenas, community centers, and playgrounds) as public health measures in Ontario ease and society adjusts to new parameters and norms. Moreover, future investigations are needed to assess if parents’ perspectives of their children’s play/sport during and following COVID-19 change as vaccines for children under 12 years become available.

## Notes

1. Ontario’s re-opening framework during this study included 3 phases; Phase 1—protect and support; Phase 2—restart; and Phase 3—recovery. Cities were assigned a phase based on number of positive COVID-19 cases in the community. Sports and/or community centers were only permitted to open in phase 2.
